# Papillary thyroid carcinoma with Hashimoto's thyroiditis: a clinical image

**DOI:** 10.11604/pamj.2026.53.139.51381

**Published:** 2026-03-24

**Authors:** Shubham Kalode, Punam Sawarkar

**Affiliations:** 1 Department of Panchakarma, Mahatma Gandhi Ayurved College, Hospital and Research Centre, Datta Meghe Institute of Higher Education and Research, Salod (H), Wardha, Maharashtra, India

**Keywords:** Papillary thyroid carcinoma, Hashimoto’s thyroiditis, clinical image

## Image in medicine

Papillary thyroid carcinoma is the most common malignant tumor of the thyroid gland and is frequently associated with chronic lymphocytic (Hashimoto´s) thyroiditis. A 45-year-old female presented with a gradually progressive anterior neck swelling of six months´ duration. There was no associated pain, dysphagia, dyspnea, or change in voice. Clinical examination revealed a firm, non-tender thyroid nodule without palpable cervical lymphadenopathy. Ultrasonography of the neck revealed a hypoechoic thyroid lesion with irregular margins. Fine needle aspiration cytology was suggestive of malignancy. Thyroidectomy was performed. Gross examination showed a firm grey-white, ill-defined lesion within the thyroid parenchyma. Histopathological examination demonstrated papillary architecture with fibrovascular cores lined by tumor cells exhibiting nuclear clearing, overlapping nuclear grooves, and occasional intranuclear cytoplasmic inclusions. The surrounding thyroid tissue showed dense lymphoplasmacytic infiltration with formation of lymphoid follicles, consistent with Hashimoto´s thyroiditis. The patient underwent complete surgical excision of the tumor. The postoperative course was uneventful, and the patient was advised to undergo regular follow-up for further oncological management.

**Figure 1:**
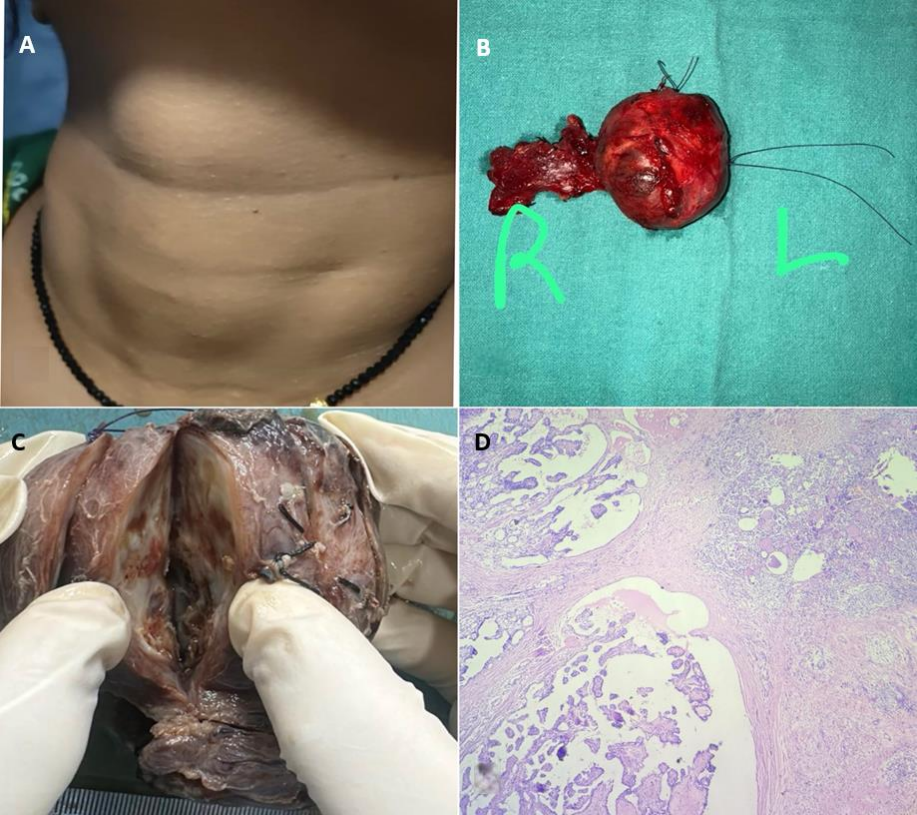
A) clinical photograph showing anterior neck swelling due to thyroid enlargement; B) gross thyroidectomy specimen showing an ill-defined firm nodular lesion (L- left lobe of thyroid, R- right lobe of thyroid); C) cut surface of thyroid gland revealing loss of normal architecture with firm infiltrative lesion; D) photomicrograph (H&E, low power) showing papillary structures with fibrovascular cores and surrounding dense lymphoid infiltration consistent with Hashimoto's thyroiditis

